# Functional involvement of γ-secretase in signaling of the triggering receptor expressed on myeloid cells-2 (TREM2)

**DOI:** 10.1186/s12974-016-0479-9

**Published:** 2016-01-20

**Authors:** Konstantin Glebov, Patrick Wunderlich, Ilker Karaca, Jochen Walter

**Affiliations:** Department of Neurology, University of Bonn, Sigmund-Freud-Str. 25, 53127 Bonn, Germany; Department of Psychiatry and Psychotherapy, University of Bonn, 53127 Bonn, Germany

**Keywords:** Triggering receptor expressed on myeloid cells-2, TREM2, γ-Secretase, Presenilin, Receptor shedding, Phosphatidylinositol-4,5-bisphosphate, Intracellular Ca^2+^, Phagocytosis

## Abstract

**Background:**

Triggering receptor expressed on myeloid cells-2 (TREM2) exerts important functions in the regulation of monocytes, like dendritic cells, osteoclasts, tissue macrophages, and microglia. Mutations in TREM2 are associated with several diseases, including Nasu-Hakola disease, frontotemporal dementia, and Alzheimer’s disease (AD). TREM2 undergoes sequential proteolytic processing by ectodomain shedding and intramembrane proteolysis.

**Findings:**

We show that inhibition of γ-secretase-dependent cleavage of the TREM2 C-terminal fragment in cellular membranes interferes with TREM2-dependent signaling and cellular function. Inhibition of γ-secretase decreases membrane-proximal signaling and intracellular Ca^2+^ response. Decreased signaling alters morphological changes and phagocytic activity of cells upon selective stimulation of TREM2.

**Conclusions:**

The data demonstrate the importance of γ-secretase-dependent intramembrane processing in TREM2-mediated signaling and, thus, a functional relation of two AD-associated proteins.

**Electronic supplementary material:**

The online version of this article (doi:10.1186/s12974-016-0479-9) contains supplementary material, which is available to authorized users.

## Findings

The triggering receptor expressed on myeloid cells-2 (TREM2) belongs to the immunoglobulin superfamily of cell surface receptors and is expressed on monocyte-derived cell types, including dendritic cells [1], osteoclasts, tissue macrophages, and microglia [2]. TREM2 associates with its co-receptor TYROBP/DNAX-activating protein of 12 kDa (DAP12). Upon activation of TREM2, DAP12 undergoes phosphorylation at two tyrosine residues within its immunoreceptor tyrosine activation motif (ITAM). Phosphorylated DAP12 recruits and activates the Syk protein kinase which mediates mitogen-activated protein kinase (MAPK) and phospholipase Cγ (PLCγ)-dependent signaling pathways [3]. TREM2 has been involved in the regulation of phagocytosis and cytokine secretion, suggesting a role of TREM2 in clearance of neuronal debris and neuroinflammation [4–6].

Mutations in either TREM2 or DAP12 can cause polycystic lipomembranous osteodysplasia with sclerosing leukoencephalopathy (PLOSL or Nasu-Hakola disease), which is also characterized by presenile dementia in homozygous carriers [7, 8]. Interestingly, TREM2 variants have recently been associated with late-onset Alzheimer’s disease (AD) and frontotemporal lobe dementia (FTD) [8–10]. Notably, TREM2 was found to be upregulated in microglia or myeloid cells surrounding characteristic amyloid plaques in mouse models of AD [11–13]. The role of TREM2 in the pathogenesis of AD remains to be determined, and studies with transgenic mouse models showed controversial effects. While deletion of TREM2 increases Aβ plaque load in one transgenic mouse model of AD [14], another study rather revealed decreased Aβ plaques in the hippocampus upon deletion of TREM2 [15]. TREM2 undergoes sequential proteolytic processing by ectodomain shedding and intramembranous cleavage by γ-secretase [16–18]. Here, we assessed the functional relevance of γ-secretase in membrane-proximal signaling of TREM2.

### Inhibition of γ-secretase impairs TREM2-dependent Ca^2+^ signaling

**Fig. 1:**
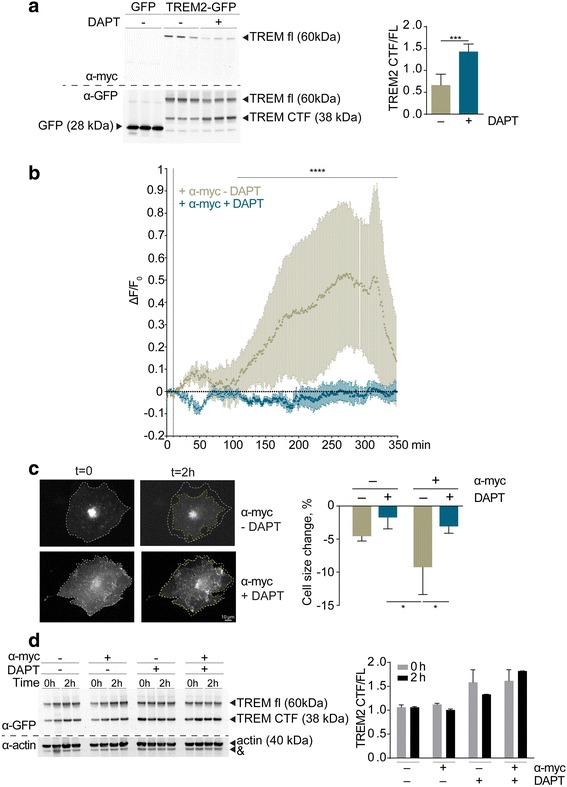
Inhibition of γ-secretase impairs TREM2-dependent Ca^2+^ signaling. **a** COS7 cells were transfected with mouse myc-TREM2-FL-GFP or control vector encoding GFP only. Constructs encoding TREM2 and DAP12 have been described earlier [18]. TREM2 was always co-transfected with DAP12 to allow efficient subcellular transport [15, 22]. Expression of TREM2-FL and TREM2-CTF was analyzed by Western immunoblotting using either anti-myc or anti-GFP antibodies. The γ-secretase inhibitor DAPT (1 μM, 18 h pretreatment) significantly increased the ratio of TREM2-CTF to TREM2-FL (***p < 0.001; *n* = 3). **b** Ratiometric determination of intracellular Ca^2+^ levels upon anti-myc antibody induced TREM2 activation in the absence or presence of DAPT (1 μM, 18 h pretreatment). COS7 cell treatment with DAPT attenuates the TREM2-evoked increase in intracellular [Ca^2+^] (****p < 0.0001; *n* = 4–6). **c** Morphological change upon activation of TREM2 in the absence or presence of DAPT. COS7 cells co-expressing mouse myc-TREM2-GFP and DAP12 were seeded onto coverslips and monitored by light microscopy for 10 min to determine spontaneous changes of the cell area. After 10 min, monoclonal anti-myc antibodies were added to the medium and cells were imaged for additional 2 h. Changes in cell surface area were determined by subtraction of the area after 2 h of stimulation from that before stimulation. Activation of TREM2 with anti-myc antibody induced a significant decrease in cell size (*p < 0.05). The inhibition of γ-secretase with DAPT prevented the TREM2-induced shrinkage of cells (*p < 0.05; *n* = 3). **d** COS7 cells were transfected with mouse myc-TREM2-FL-GFP and treated with anti-myc antibody for 2 h after overnight incubation with or without DAPT. TREM2 FL and CTFs were detected by western immunobloting

TREM2 signaling involves the activation of PLCγ and elevation of the cytosolic Ca^2+^ concentration [14, 18]. To investigate the role of γ-secretase in TREM2-dependent signaling, we expressed a TREM2 variant with an N-terminal myc tag and a C-terminal green fluorescent protein (GFP) tag together with its co-receptor DAP12. We previously showed that myc-tagged TREM2 could be specifically activated by binding of anti-myc antibodies leading to phosphorylation of its co-receptor DAP12. The C-terminal GFP tag allowed the identification of transfected cells but did not interfere with the constitutive proteolytic processing of TREM2 [18]. Western immunoblotting with anti-myc and anti-GFP antibodies revealed expression of full-length TREM2 with the N- and C-terminal tags at ~60 kDa (Fig. 1a). In addition, a ~38 kDa band was selectively detected with the anti-GFP antibody representing a C-terminal fragment (CTF) of TREM2 after ectodomain shedding (Fig. 1a). Pharmacological inhibition of γ-secretase with DAPT (*N*-[(3,5-difluorophenyl)acetyl]-l-alanyl-2-phenylglycine-1,1-dimethylethyl ester) led to the accumulation of the TREM2 CTF, thereby significantly increasing the ratio of CTF/full-length TREM2 (Fig. 1a).

Our previous data revealed that inhibition of γ-secretase decreased the phosphorylation of the TREM2 co-receptor DAP12 and stabilized PIP2 levels at the plasma membrane, suggesting impaired activation of PLCγ [18]. Because PLCγ plays a major role in the regulation of intracellular Ca^2+^ signaling, we next analyzed the effect of γ-secretase inhibition on TREM2-dependent changes in intracellular Ca^2+^ concentrations ([Ca^2+^]_i_). TREM2 was specifically stimulated with anti-myc antibody, and changes in [Ca^2+^]_i_ were monitored by Fluo-4 AM (Life Technologies, USA) imaging. Cell incubation with anti-myc caused a significant increase in cytosolic [Ca^2+^]_i_ (Fig. 1b). An isotype control antibody did not evoke a signal (not shown), indicating that specific activation of TREM2 results in elevation of [Ca^2+^]_i_. Interestingly, pharmacological inhibition of γ-secretase with DAPT efficiently blocked the elevation of [Ca^2+^]_i_ upon activation of TREM2 (Fig. 1b).

It has been shown previously that activation of TREM2 induces changes in cellular morphology [19]. Thus, we next tested morphological changes of cells upon activation of TREM2 in the presence or absence of γ-secretase inhibition. Cells expressing myc-tagged TREM2 were monitored by live cell microscopy in the presence of anti-myc or isotype control antibodies. Cell incubation with anti-myc antibody, but not with the isotype control (data not shown), resulted in a time-dependent decrease in cell surface area (Fig. 1c). Notably, inhibition of γ-secretase completely blocked the TREM2-dependent changes in cellular morphology (Fig. 1c).

To test whether the addition of anti-myc antibody to living cells affects the processing of TREM2 under the experimental conditions, we analyzed levels of full-length TREM2 and its CTFs by Western immunoblotting. However, treatment with anti-myc antibody did not affect the generation of TREM2-CTF (Fig. 1d).

### Decreased TREM2-dependent phagocytosis upon inhibition of γ-secretase activity

**Fig. 2:**
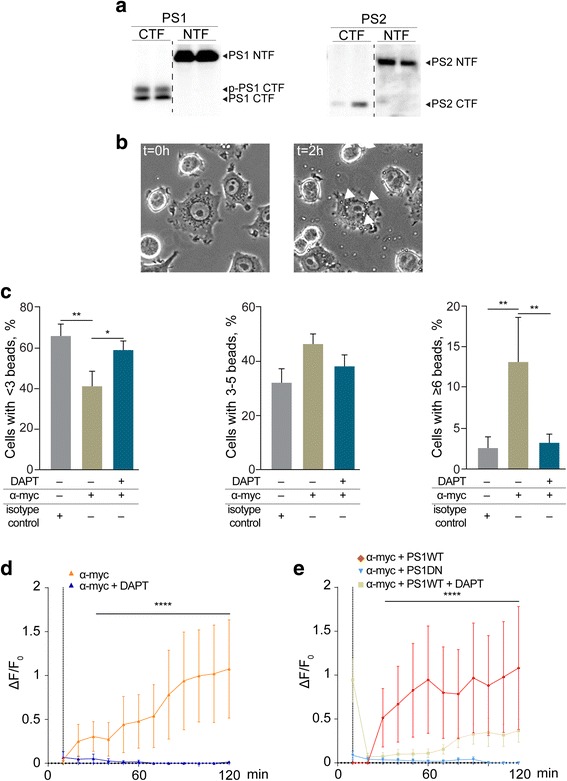
Inhibition of γ-secretase activity decreases TREM2-dependent phagocytosis in microglial BV-2 cells. **a** Analysis of endogenous γ-secretase components PS1 and PS2 in microglial BV-2 cells by Western immunoblotting. Characteristic N-terminal fragment (*NTF*) and C-terminal fragment (*CTF*) of PS1 and PS2 were detected, indicating endogenous expression of both PS proteins. P-PS1 CTF denotes the migration of a phosphorylated variant of the PS1 CTF. **b**, **c** BV-2 cells were seeded on μ-IBIDI dishes (IBIDI, Germany) and transfected with myc-TREM2-GFP. Cells were cultured in the absence or presence of 1 μM of DAPT for 24 h. Four microliters of latex beads (Sigma-Aldrich, USA) was added, and TREM2 was stimulated for 2 h by addition of anti-myc antibody. Uptake of latex beads was analyzed by light microscopy (*p < 0.05; **p < 0.01; *n* = 97–105 cells per condition). *Arrow heads* indicate internalized leads. **d** BV-2 cells with endogenous expression of PS1 and PS2 (see **a**) were preincubated in the absence or presence of DAPT (**d**) for 24 h. Cells were stimulated with anti-myc antibodies, and changes in [Ca^2+^]_i_ were monitored by fluorescence microscopy. Treatment with DAPT abrogated the cellular response in [Ca^2+^]_i_. **e** Cells stably overexpressing PS1 wild type (*PS1 WT*) or a dominant-negative variant of PS1 (*PS1 DN*) were stimulated with anti-myc. Only cells expressing PS1 WT, but not PS1 DN, responded to TREM2 activation with an increase in [Ca^2+^]_i_. As observed in cells with endogenous PS expression, pretreatment of PS1 WT-overexpressing cells with DAPT also inhibited the increase in [Ca^2+^]_i_ (****p < 0.0001; *n* = 3 to 11 cells per condition)

TREM2 is predominantly expressed in monocyte-derived cells, including macrophages and microglia, and modulates phagocytosis [2]. However, functional investigation of endogenous TREM2 is hampered by the lack of a selective ligand for TREM2. We adapted our heterologous expression system to the mouse microglial cell line BV-2. BV-2 cells endogenously express functional PS1 and PS2 proteins as indicated by the detection of characteristic N-terminal and C-terminal fragments derived from endo-proteolytic processing of the full-length proteins during incorporation and maturation of the γ-secretase complex (Fig. 2a). In contrast, endogenous TREM2 could not be detected by Western immunoblotting (not shown).

BV-2 cells were transfected with the myc-tagged variant of TREM2 as described above, and phagocytosis of latex beads was assessed by live cell microscopy upon incubation for 2 h in the absence or presence of antibodies against the myc epitope to selectively activate TREM2 (Fig. 2b, c; Additional file 1: Movie 1). The fraction of cells with less than three beads was significantly lower upon activation with anti-myc antibody (40.82 ± 7.63 %) in comparison to cells treated with an isotype control antibody (65.83 ± 5.78 %). This effect was not observed after pretreatment of cells with the γ-secretase inhibitor DAPT (58.80 ± 4.62 %; Fig. 2c). The fraction of cells with three to five internalized beads slightly increased from 32.01 ± 5.21 % in cultures incubated with an isotype control antibody to 46.12 ± 3.96 % in cultures incubated with the anti-myc antibody (Fig. 2c). However, this effect was not statistically significant. The fraction of cells which have internalized six or more beads, thus reflecting high phagocytic activity, significantly increased from 2.55 ± 1.41 % (isotype control) to 13.06 ± 5.50 % upon specific activation of TREM2 (with anti-myc antibody), representing an aproximatley fivefold stimulation (Fig. 2c). These data indicate that selective activation of TREM2 stimulates the phagocytic activity of BV-2 cells. Interestingly, inhibition of γ-secretase activity by preincubation of cells with DAPT significantly decreased the TREM2-mediated stimulation of phagocytosis to basal levels of cells incubated with isotype control antibodies (Fig. 2c).

We next assessed effects of γ-secretase inhibition on Ca^2+^ signaling in BV-2 cells. As described above for COS7 cells, stimulation of TREM2 overexpressing BV-2 cells resulted in increased [Ca^2+^]_i_. Inhibition of γ-secretase by DAPT strongly inhibited the elevation of [Ca^2+^]_i_ (Fig. 2d). We also used a genetic approach to inhibit γ-secretase activity (Fig. 2e). BV-2 cells were stably transfected with a dominant-negative variant of PS1 (PS1 DN), which has a mutation in its active site and thus is inactive [18]. Since this variant associated with other components of the γ-secretase complex, it suppresses endogenous γ-secretase activity [18]. Cells stably overexpressing the wild-type PS1 (PS1 WT) were used as control. Activation of myc-tagged TREM2 in PS1 WT-overexpressing cells increased [Ca^2+^]_i_. In contrast, cells expressing the PS1 DN did not respond to stimulation of TREM2. The response of PS1 WT-expressing cells was also decreased upon pharmacological γ-secretase inhibition with DAPT (Fig. 2e).

The present data revealed that γ-secretase activity is important to control the signaling function of TREM2. The physiological functions of TREM2 are not fully understood. On the one hand, macrophages of TREM2 knockout (KO) mice show increased production of inflammatory cytokines upon Toll-like receptor activation [20] and decreased phagocytic activity [15]. On the other hand, activation of TREM2 in microglia increased the expression of chemokine receptors and cell migration [21, 22]. Thus, TREM2 could exert complex functions in cellular activation. Interestingly, homozygous mutations in TREM2 have been shown to cause Nasu-Hakola disease and FTD, while the heterozygous R47H variant is associated with an increased risk for development of AD [11, 16, 23]. Several of these disease-associated mutations impair transport in the secretory pathway and result in lower glycosylation and expression at the cell surface [16, 17]. HEK293 or BV-2 cells overexpressing mutant TREM2 showed decreased phagocytic activity as compared to cells expressing WT TREM2, suggesting that the mutations of TREM2 cause a loss of function [16]. Recent data also showed that the deletion of TREM2 led to decreased clustering of microglia around amyloid plaques in a mouse model of AD and also decreased the phagocytic activity [14]. Another study also showed decreased number of myeloid cells in brains of TREM2 KO mice crossed with APP transgenic mice [15]. However, cells associated with plaques in this model revealed characteristics of peripheral macrophages that might invade the brain [15].

**Fig. 3:**
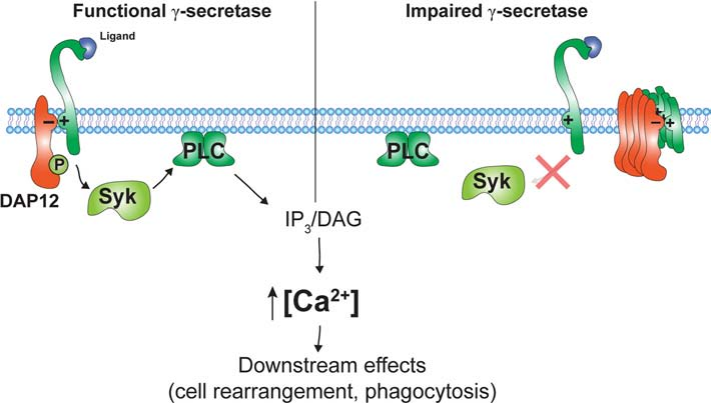
Hypothetical model of TREM2-dependent signaling in cells with and without functional γ-secretase. Degradation of TREM2 CTFs by γ-secretase allows an efficient association of full-length TREM2 with its co-receptor DAP12. Upon ligand binding to TREM2, DAP12 undergoes phosphorylation within its intracellular ITAM domain, resulting in the recruitment of the spleen tyrosine kinase (*Syk*) and activation of phospholipase C (PLC). PLC-mediated cleavage of phosphatidylinositol-4,5-bisphosphate in cellular membranes leads to generation of inositol-1,4,5-trisphosphate (IP_3_) and mobilization in Ca^2+^ from the endoplasmic reticulum, thereby regulating phagocytosis and dynamics of the cytoskeleton. Upon inhibition of γ-secretase, DAP12 might predominantly associate with accumulated TREM2 CTFs, thereby decreasing the availability to bind to full-length TREM2, which would result in impaired signal transduction

Our present data revealed that inhibition of γ-secretase activity also decreased TREM2-dependent signaling and phagocytosis. A role of γ-secretase in microglial phagocytosis and migration has been shown previously. Isolated microglia from PS2 KO mice showed decreased phagocytosis and migration [24, 25], but the molecular mechanisms were unclear. The present data could suggest that impaired processing of the TREM2 CTF by γ-secretase might contribute to these previously observed effects. The signaling of TREM2 requires interaction with its co-receptor DAP12. We previously showed that the accumulation of TREM2 CTFs alters the distribution and association of DAP12 with full-length TREM2 at the plasma membrane [18]. Together with the present data, these findings suggest that accumulated TREM2 CTFs might trap DAP12 and thereby interfere with TREM2 signaling (Fig. 3).

A recent study showed that DAP12 overexpression could stabilize TREM2 CTFs [25]. In turn, the overexpression of TREM2 CTF impaired the secretion of inflammatory cytokines upon cell exposure to lipopolysaccharide, also supporting the notion that efficient clearance of CTFs is critical for proper signaling function of TREM2 [25].

The present data demonstrate an important function of γ-secretase activity in the signal transduction of TREM2. The inhibition of γ-secretase impaired Ca^2+^ signaling and phagocytic activity of TREM2-expressing cells. Thus, these findings support a functional involvement of two critical genetic factors of AD in the same signaling pathway. The functional connection of γ-secretase and TREM2 should be considered in the pharmacological targeting of γ-secretase in the treatment of AD.

## Additional file

Additional file 1:
**Phagocytosis of latex beads by BV-2 cells.** (MP4 33426 kb)

## Abbreviations

AD: Alzheimer’s disease
CTF: C-terminal fragment
DAP12: DNAX-activating protein of 12 kDa
DAPT: *N*-[(3,5-difluorophenyl)acetyl]-l-alanyl-2-phenylglycine-1,1-dimethylethyl ester
GFP: green fluorescent protein
PLC: phospholipase C
TREM2: triggering receptor expressed on myeloid cells-2

## References

[1] Bouchon A, Hernandez-Munain C, Cella M, Colonna M (2001). A DAP12-mediated pathway regulates expression of CC chemokine receptor 7 and maturation of human dendritic cells. J Exp Med.

[2] Colonna M (2003). TREMs in the immune system and beyond. Nat. Rev. Imm.

[3] Lowell CA. Src-family and Syk kinases in activating and inhibitory pathways in innate immune cells: signaling cross talk. Cold Spring Harb Perspect Biol. 2011;3(3). doi: 10.1101/cshperspect.a002352. 10.1101/cshperspect.a002352 PMC3039931 21068150

[4] Lue LF, Schmitz C, Walker DG. What happens to microglial TREM2 in Alzheimer’s disease: immunoregulatory turned into immunopathogenic? Neuroscience. 2014. doi: 10.1016/j.neuroscience.2014.09.050 10.1016/j.neuroscience.2014.09.050 25281879

[5] Kawabori M, Kacimi R, Kauppinen T, Calosing C, Kim JY, Hsieh CL (2015). Triggering receptor expressed on myeloid cells 2 (TREM2) deficiency attenuates phagocytic activities of microglia and exacerbates ischemic damage in experimental stroke. J Neurosci.

[6] Hickman SE, El Khoury J (2014). TREM2 and the neuroimmunology of Alzheimer’s disease. Biochem Pharmacol.

[7] Paloneva J, Kestila M, Wu J, Salminen A, Bohling T, Ruotsalainen V (2000). Loss-of-function mutations in TYROBP (DAP12) result in a presenile dementia with bone cysts. Nat Genet.

[8] Guerreiro R, Wojtas A, Bras J, Carrasquillo M, Rogaeva E, Majounie E (2013). TREM2 variants in Alzheimer’s disease. N Engl J Med.

[9] Jonsson T, Stefansson H, Steinberg S, Jonsdottir I, Jonsson PV, Snaedal J (2013). Variant of TREM2 associated with the risk of Alzheimer’s disease. N Engl J Med.

[10] Guerreiro RJ, Lohmann E, Bras JM, Gibbs JR, Rohrer JD, Gurunlian N (2013). Using exome sequencing to reveal mutations in TREM2 presenting as a frontotemporal dementia-like syndrome without bone involvement. JAMA Neurol.

[11] Guerreiro R, Hardy J (2013). TREM2 and neurodegenerative disease. N Engl J Med.

[12] Frank S, Burbach GJ, Bonin M, Walter M, Streit W, Bechmann I (2008). TREM2 is upregulated in amyloid plaque-associated microglia in aged APP23 transgenic mice. Glia.

[13] Melchior B, Garcia AE, Hsiung BK, Lo KM, Doose JM, Thrash JC (2010). Dual induction of TREM2 and tolerance-related transcript, Tmem176b, in amyloid transgenic mice: implications for vaccine-based therapies for Alzheimer’s disease. ASN Neuro.

[14] Wang Y, Cella M, Mallinson K, Ulrich JD, Young KL, Robinette ML (2015). TREM2 lipid sensing sustains the microglial response in an Alzheimer’s disease model. Cell.

[15] Jay TR, Miller CM, Cheng PJ, Graham LC, Bemiller S, Broihier ML (2015). TREM2 deficiency eliminates TREM2+ inflammatory macrophages and ameliorates pathology in Alzheimer’s disease mouse models. J Exp Med.

[16] Kleinberger G, Yamanishi Y, Suarez-Calvet M, Czirr E, Lohmann E, Cuyvers E et al. TREM2 mutations implicated in neurodegeneration impair cell surface transport and phagocytosis. Sci Transl Med. 2014;6(243):243ra86. doi: 10.1126/scitranslmed.3009093. 10.1126/scitranslmed.3009093 24990881

[17] Park JS, Ji IJ, An HJ, Kang MJ, Kang SW, Kim DH (2015). Disease-associated mutations of TREM2 alter the processing of N-linked oligosaccharides in the Golgi apparatus. Traffic.

[18] Wunderlich P, Glebov K, Kemmerling N, Tien NT, Neumann H, Walter J (2013). Sequential proteolytic processing of the triggering receptor expressed on myeloid cells-2 (TREM2) protein by ectodomain shedding and gamma-secretase-dependent intramembranous cleavage. J Biol Chem.

[19] Kiialainen A, Veckman V, Saharinen J, Paloneva J, Gentile M, Hakola P (2007). Transcript profiles of dendritic cells of PLOSL patients link demyelinating CNS disorders with abnormalities in pathways of actin bundling and immune response. J Mol Med (Berl).

[20] Turnbull IR, Gilfillan S, Cella M, Aoshi T, Miller M, Piccio L (2006). Cutting edge: TREM-2 attenuates macrophage activation. J Immunol.

[21] Takahashi K, Prinz M, Stagi M, Chechneva O, Neumann H (2007). TREM2-transduced myeloid precursors mediate nervous tissue debris clearance and facilitate recovery in an animal model of multiple sclerosis. PLoS Medicine.

[22] Takahashi K, Rochford CD, Neumann H (2005). Clearance of apoptotic neurons without inflammation by microglial triggering receptor expressed on myeloid cells-2. J Exp Med.

[23] Soragna D, Papi L, Ratti MT, Sestini R, Tupler R, Montalbetti L (2003). An Italian family affected by Nasu-Hakola disease with a novel genetic mutation in the TREM2 gene. J Neurol Neurosurg Psychiatry.

[24] Lee J, Chan SL, Mattson MP (2002). Adverse effect of a presenilin-1 mutation in microglia results in enhanced nitric oxide and inflammatory cytokine responses to immune challenge in the brain. Neuromolecular Med.

[25] Zhong L, Chen XF, Zhang ZL, Wang Z, Shi XZ, Xu K et al. DAP12 stabilizes the C-terminal fragment of the triggering receptor expressed on myeloid cells-2 (TREM2) and protects against LPS-induced pro-inflammatory response. J Biol Chem. 2015. doi: 10.1074/jbc.M115.645986. 10.1074/jbc.M115.645986 PMC4505493 25957402

